# Tissue Anisotropy Modeling Using Soft Composite Materials

**DOI:** 10.1155/2018/4838157

**Published:** 2018-05-09

**Authors:** Arnab Chanda, Christian Callaway

**Affiliations:** ^1^Department of Aerospace Engineering, University of Alabama, Tuscaloosa, AL 35401, USA; ^2^Department of Bioengineering, University of Pittsburgh, Pittsburgh, PA 15213, USA

## Abstract

Soft tissues in general exhibit anisotropic mechanical behavior, which varies in three dimensions based on the location of the tissue in the body. In the past, there have been few attempts to numerically model tissue anisotropy using composite-based formulations (involving fibers embedded within a matrix material). However, so far, tissue anisotropy has not been modeled experimentally. In the current work, novel elastomer-based soft composite materials were developed in the form of experimental test coupons, to model the macroscopic anisotropy in tissue mechanical properties. A soft elastomer matrix was fabricated, and fibers made of a stiffer elastomer material were embedded within the matrix material to generate the test coupons. The coupons were tested on a mechanical testing machine, and the resulting stress-versus-stretch responses were studied. The fiber volume fraction (FVF), fiber spacing, and orientations were varied to estimate the changes in the mechanical responses. The mechanical behavior of the soft composites was characterized using hyperelastic material models such as Mooney-Rivlin's, Humphrey's, and Veronda-Westmann's model and also compared with the anisotropic mechanical behavior of the human skin, pelvic tissues, and brain tissues. This work lays the foundation for the experimental modelling of tissue anisotropy, which combined with microscopic studies on tissues can lead to refinements in the simulation of localized fiber distribution and orientations, and enable the development of biofidelic anisotropic tissue phantom materials for various tissue engineering and testing applications.

## 1. Introduction

Soft tissues in the human body, namely, the skin, skeletal muscles, connective tissues, and tissues forming the organs (such as the brain and myocardial tissues), are not homogeneous or isotropic [1, 2]. These tissues exhibit regional and directional anisotropy in three-dimensional space [3]. This material anisotropy could be mainly attributed to the variations in the distribution of collagen fibers in tissues [2]. In the past, collagen fiber distribution in human cadavers and animal models was studied using histological investigations [4, 5]. Advancements in imaging techniques in recent years have allowed looking at fiber distributions in the human body using the diffusion tensor magnetic resonance imaging (DT-MRI) technique [6]. However, recreating such fiber-tissue model in a computational framework is challenging due to four reasons. First, the fibers are in the form of lines or splines in a DT-MRI model, which needs to be converted to volumes before they could be integrated with a tissue matrix volume. Second, a huge percentage of fibers overlap with each other making it very difficult to generate clean fiber meshes which could be used in analyses. Third, the exact number of fibers in a region is difficult to estimate, unless a histological study is conducted for that region to calculate the fiber volume fraction (FVF). Fourth, most tissues continuously merge with other tissues with no discernible boundaries (e.g., it is difficult to capture the left ventricular (LV) tissues in the heart as it transitions into the right ventricle (RV) and other sections of the heart [7]).

In the literature, there have been few attempts to incorporate tissue anisotropy in finite element (FE) models [1, 3, 7–12]. The most common method has been to discretize a tissue or an organ into regions (with a discernible fiber orientation) and approximate a principal fiber direction in those regions. A stiffer material property is assigned in the principal fiber direction compared to the other directions [1, 8], which can also be loaded using various passive excitation methods [10]. Another method has been to model a tissue region using transversely isotropic material formulation [13, 14].

Tissue anisotropy was incorporated successfully computationally in various recent FE models. Chanda et al. in 2011 developed a female pelvic system model with an anisotropic levator ani (LA) muscle [3] and simulated the different stages of labor. The LA muscle was decomposed into multiple sections, and the principal fiber directions in each of these sections were identified. To induce anisotropy, the relative stiffness between the fiber and the matrix components was varied while maintaining the same overall stress-versus-strain response along the fiber direction. Two pelvic floor models were developed with different anisotropy ratios for the LA muscle, and a fetal skull model was made to pass through the vaginal canal and pelvic floor muscles including the LA. The analyses revealed that by increasing the fiber anisotropy, the mechanical response recorded for a LA muscle is significantly affected along with a decrease in the magnitude of force required for delivery. No validation techniques were, however, adopted due to the lack of experimental data. Researchers working on traumatic brain injury (TBI) have recently used tissue anisotropy material modeling techniques to advance the biofidelity and precision of the numerical computations [11]. Mainly from a tissue anisotropy perspective, the white matter of the brain was being looked at due to the coherent orientation of fibers [15]. Weiss et al. [16] segmented the human left ventricle (LV) into small cuboidal segments and recorded the myocardial fiber distributions in each of these segments. Finite element simulations were conducted to study the influence of fiber orientations on the activation sequence of the various sections of the LV, and the changes at each of the segments were tracked dynamically. Chanda et al. in 2015 [7] modeled the anisotropy in heart tissues by using the helical ventricular myocardial band (HVMB) theory [17]. The two layers of fibers (going in different directions) in the heart were simulated, and their effect on the pumping efficiency of the heart was investigated.

Experimentally, anisotropy has been measured in skin [12, 18], pelvic [19–21], and brain tissues [22]; however, to date, there exists no tissue simulant or phantom material which incorporates tissue anisotropy. A way to physically model anisotropic tissue materials would allow the validation of the results from the computational models. Also, such a model would be indispensable to generate realistic tissue phantoms with anisotropic effects, for various biomechanical testing and tissue engineering applications. In the current work, soft composite-like materials made of elastomers were used to macroscopically simulate tissue anisotropy at the scale of tensile-testing coupons. The anisotropic mechanical behavior of the skin, pelvic, and brain tissues were compared with the properties of the soft composites. Also, the effect of varying FVF, fiber spacing, and orientation were investigated. Additionally, the nonlinear stress-versus-stretch responses of the tissue simulants were characterized using hyperelastic constitutive relationships. The following sections discuss the various methodologies for fabrication of the novel soft composites, key results, and conclusions.

## 2. Materials and Methods

### 2.1. Preparation of Matrix and Fiber Materials

**Figure 1:**
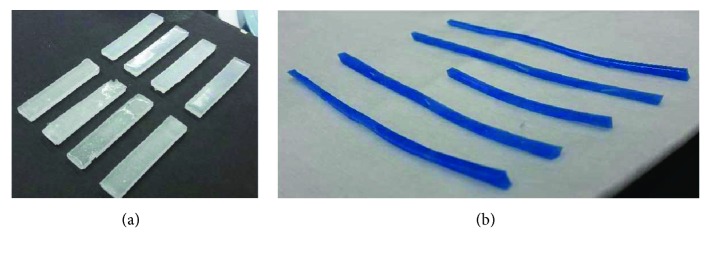
(a) Soft silicone matrix. (b) Hard silicone fibers used in fabricating the soft tissue composites.

**Figure 2:**
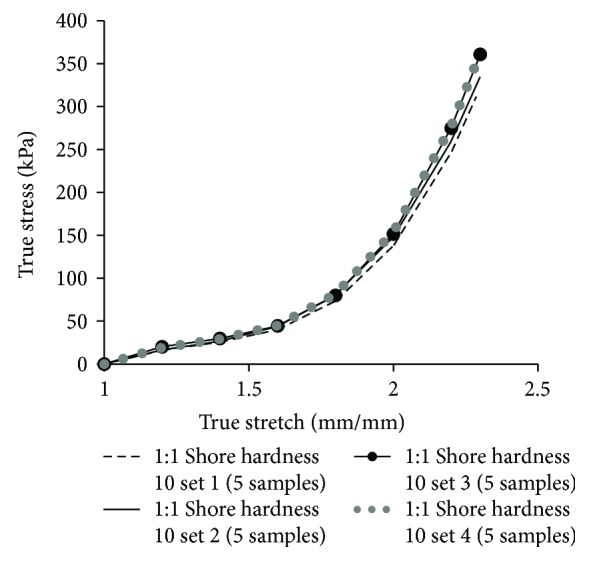
Stress-versus-stretch plots for four batches of samples (5 in each) with a 1 : 1 ratio of a two-part elastomer with a shore hardness of 10, simulating matrix material.

Elastomer-based materials are characterized using the Shore (Durometer) hardness scale, defined as per the American Society for Testing and Materials (ASTM) D2240 testing standard [23]. A two-part extremely soft elastomer material with a shore hardness of 10 was used for developing the matrix material. Part A and part B were mixed at a 1 : 1 ratio to generate 20 test coupons (Figure 1(a)) with the dimensions of 49 mm < length < 50 mm, 9 mm < width < 11 mm, and 2 mm < thickness < 3 mm. Each coupon was clamped on a universal tensile testing machine (MTS Criterion 42) and tested at a constant strain rate of 0.4 mm/s [12]. Several considerations were taken while testing the soft materials [24]. First, soft materials slip very easily, thereby special grips coated with a rubber-like material which provides high friction against slipping were used. Second, strain rate has been observed to significantly affect the load response of soft materials [25], and thus a specific strain rate was used, so that results can be precisely compared with literature. Additionally, a very small initial load (<0.1 N) was applied on each test specimen to ensure that there is no initial slack in the specimen. The stress-versus-strain plots generated from the tests were checked for repeatability (Figure 2 shows the average plots for the four sets of samples tested) and also compared with the literature [26–29] to ensure no machine calibration errors.

**Figure 3:**
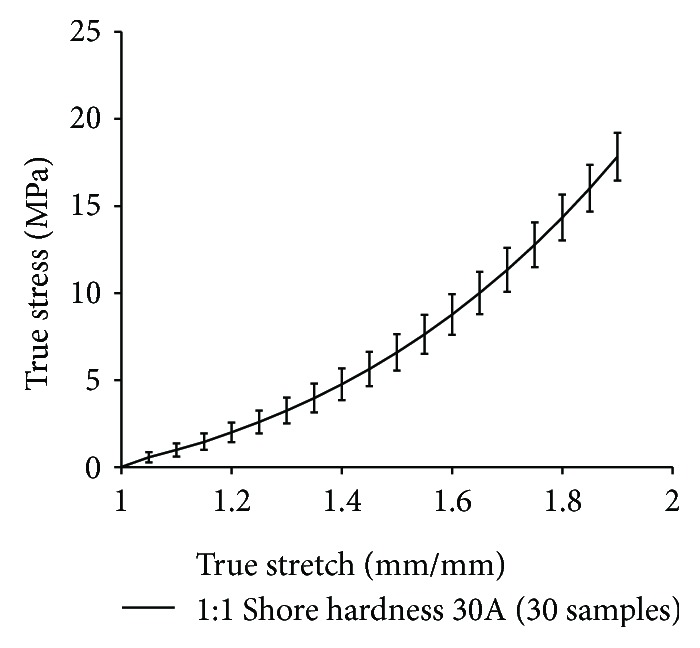
Stress-versus-stretch plots of 30 coupons with a 1 : 1 ratio of a two-part elastomer with a shore hardness of 30A, simulating fiber material.

A two-part hard elastomer material with a shore hardness of 30A was selected to make the hard fibers (Figure 1(b)). 30 test coupons were generated using a 1 : 1 ratio of part A and part B of the elastomer and tested mechanically on the MTS machine. The combined stresses-versus-stretch results for the 30 specimens are presented in Figure 3. Hard silicone sheets with an area of 210 mm × 297 mm and a thicknesses of 2 mm and 4 mm, respectively, were fabricated, and multiple thin fibers were cut with different lengths and widths. The following section discusses the fabrication of the composite material using the soft matrix material and the hard fiber material.

### 2.2. Soft Tissue Composite Fabrication

**Figure 4:**
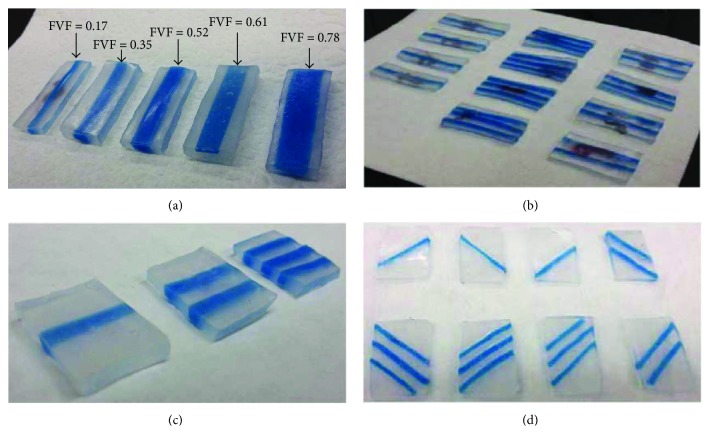
Composite test specimens with (a) varying fiber volume fractions (FVF), (b) one, two, or three similar fibers, (c) transverse fibers, and (d) skewed fibers at ±45°.

Fibers made of the hard elastomer material and of different widths and thicknesses were laid in a rectangular box, and the soft elastomer material was poured into it. Test specimens were cut out with the following dimensions: length 30 ± 3 mm, width 10 ± 4 mm, and thickness 2 ± 2 mm. The fiber volume fraction (FVF) for each of the test specimens was calculated based on (1). The lowest and highest FVFs were estimated to be 0.17 and 0.78, respectively.  Figure 4(a) shows the range of specimens created with different FVFs for further testing.

(1) $$\begin{array}{c} Fiber\;volume\;fraction\;\left(FVF\right)=\frac{fiber\;volume}{total\;specimen\;volume}. \end{array}$$

Multiple fibers were cut with similar cross sections (with 2 mm < width < 3 mm and 2 mm < thickness < 3 mm), and test specimens were fabricated with single, two, and three fibers (Figure 4(b)). The effect of variations of the fiber spacing and number on soft composite mechanical properties were investigated. The overall dimensions of the specimens with one, two, and three fibers were 35 mm^∗^10 mm^∗^3 mm, 35 mm^∗^15 mm^∗^3 mm, and 35 mm^∗^18 mm^∗^3 mm, respectively. Each specimen was tested with the fibers pointing in the longitudinal direction (along the line of the force). Also, it was made sure that the ends of all the fibers in each specimen were gripped in clamps. It should be mentioned that in one of the specimen, a fiber was 4 degrees off from the longitudinal direction, which was assumed to be acceptable for the study.

In another form, soft composite specimens were cut out with the fibers pointing in the direction transverse to the direction of load application. Also, different variants had one, two, and three fibers (Figure 4(c)). A total of 10 such specimens with 20 mm < length < 25 mm, 15 < width < 18 mm, and 3 mm < thickness < 4 mm were fabricated and tested on the mechanical testing machine.

Skewed fibers were placed at ±45 degrees, and 3 specimens with one, two, and three fibers, respectively, were fabricated (Figure 4(d)). The specimens were not of the same size, and the dimension ranges were with 25 mm < length < 35 mm, 15 mm < width < 20 mm, and 2 mm < thickness < 3 mm. Each specimen was clamped in such a way that the fiber direction was at ±45 degrees relative to the direction of the application of the load.

All the test specimens were tested at a constant strain rate of 0.4 mm/s, and raw data was obtained from the mechanical testing machine in the form of load-versus-extension data points. The maximum crosshead distance allowed for the tests was 50 mm. For postprocessing of the raw load-extension curves, a well-defined protocol was followed comprising of seven major steps. First, any part of the plots which show negative load values was trimmed off (which may arise from the specimen being slack initially). Second, any part of the plots after the yield point was trimmed off, as they were insignificant for our analysis. Third, the graphs were calibrated and shifted as required to start from the origin. Fourth, the engineering stress-versus-engineering strain plots were replotted as true stress-versus-true strain plots which were obtained using (2) and (3). Fifth, 3rd-degree polynomial trend lines were fitted to each of the plots with *R*^2^ (coefficient of determination) values between 0.99 and 1. Sixth, the strain (*x*-axis) intervals (0.01) and range (0 < *ε*_true_ < 1) were standardized, and the respective stress values (*y*-axis) were calculated using the trend line equations obtained in step 5, and replotted. Seventh, each standardized stress-strain plot was converted to stress-stretch (*λ*) plots using (4). The stress-stretch curves were also fit into hyperelastic material models (discussed in Section 2.3). Additionally, the biomechanical behavior of the soft composites were compared with the mechanical properties of the human skin, pelvic, and brain tissues.

(2) $$\begin{array}{c} \sigma_{true}=\sigma_{eng}*\left(1+\varepsilon_{eng}\right), \end{array}$$

(3) $$\begin{array}{c} \varepsilon_{true}=\ln\left(1+\varepsilon_{eng}\right), \end{array}$$

(4) $$\begin{array}{c} \lambda =1+\varepsilon . \end{array}$$

### 2.3. Nonlinear Material Characterization

Soft materials show a nonlinear stress-versus-stretch response, which can be characterized using hyperelastic curve fit equations such as the Fung, Mooney-Rivlin, Yeoh, Neo-Hookean, Ogden, Humphrey, Martins, or Veronda-Westmann models [24]. Hyperelastic constitutive models are based on the definition of the strain-energy function (denoted as *ψ*), which depends on the type of material [30, 31]. Any hyperelastic model is dependent on the principal stretches (*λ*_1_, *λ*_2_, and *λ*_3_) which are further dependent on the Cauchy-Green tensor invariants (*I*_1_, *I*_2_, and *I*_3_) [24] as shown in (5).

(5) $$\begin{array}{c} \psi =\psi \left(I_{1},I_{2},I_{3}\right),\\ I_{1}=\overset{3}{\underset{i=1}{\sum }}\lambda_{i}^{2},\\ I_{2}=\overset{3}{\underset{i,j=1}{\sum }}\lambda_{i}^{2}\lambda_{j}^{2},\\ I_{3}=\overset{3}{\underset{i=1}{\prod }}\lambda_{i}^{2}. \end{array}$$

In the current work, the Mooney-Rivlin, Humphrey, and Veronda-Westmann hyperelastic models were used to characterize the mechanical behavior of the soft composite materials. All these models have been used in the past to numerically predict soft tissue biomechanical behavior [24]. The strain energy functions of these three models are shown in (6).

(6) $$\begin{array}{c} \psi_{Mooney‐Rivlin}=\overset{2}{\underset{i=1}{\sum }}c_{i}\left(I_{i}-3\right),\\ \psi_{Humphrey}=c_{1}\left(e^{c_{2}\left(I_{1}-3\right)}-1\right),\\ \psi_{Veronda‐Westmann}=c_{1}\left[e^{c_{2}\left(I_{1}-3\right)}-1\right]-\frac{c_{1}c_{2}}{2}\left(I_{2}-3\right). \end{array}$$

Though uniaxial tests on the soft composite specimens and following the procedure outlined in the literature by Martins et al. [24], the principal Cauchy stress is expressed in terms of the stretch and the strain energy function using (7) and (8). Using the strain energy equations in (6), the nonlinear stress-stretch behavior of the specimens can be predicted using (9), (10), and (11) for uniaxial tests.

(7) $$\begin{array}{c} \sigma_{1}=\lambda_{1}\frac{\partial \psi }{\partial \lambda_{1}}-\lambda_{3}\frac{\partial \psi }{\partial \lambda_{3}}, \end{array}$$

(8) $$\begin{array}{c} \sigma_{2}=\sigma_{3}=0, \end{array}$$

(9) $$\begin{array}{c} \sigma_{Mooney‐Rivlin}=2\left(\lambda^{2}-\frac{1}{\lambda }\right)\left(c_{1}+c_{2}\frac{1}{\lambda }\right), \end{array}$$

(10) $$\begin{array}{c} \sigma_{Humphrey}=2\left(\lambda^{2}-\frac{1}{\lambda }\right)c_{1}c_{2}e^{c_{2}\left(I_{1}-3\right)}, \end{array}$$

(11) $$\begin{array}{c} \sigma_{Veronda‐Westmann}=2\left(\lambda^{2}-\frac{1}{\lambda }\right)c_{1}c_{2}\left(e^{c_{2}\left(I_{1}-3\right)}-\frac{1}{2\lambda }\right). \end{array}$$

In this work, true stress-versus-true stretch data obtained from the mechanical tests were fit into (9), (10), and (11) using the Microsoft Excel curve fit solver which utilizes a common GRG (generalized reduced gradient) nonlinear optimization algorithm [29, 32, 33]. Before running the solver, an initial selection of arbitrary parameters for the hyperelastic equation was conducted and used along with the stretch values (in the range of 1-2) to predict stress values. The predicted stress-versus-stretch was plotted alongside the experimental stress-versus-stretch plot. The sum of squares of differences between the actual and predicted stress values (for all stretch values) was computed, and this value was fed into the Excel curve fit solver along with the arbitrary parameters chosen. On solving, this value was minimized and the best curve fit parameters were returned by Excel. In order to ensure the accuracy of curve fitting, the predicted plot and actual plot were compared using *R*^2^ correlation value calculation in Excel. Only the hyperelastic parameters generating curve fits with 0.99 < *R*^2^ < 1 were reported.

## 3. Results and Discussion

### 3.1. Effect of Fiber Volume Fraction (FVF) on Soft Composite Mechanical Properties

**Figure 5:**
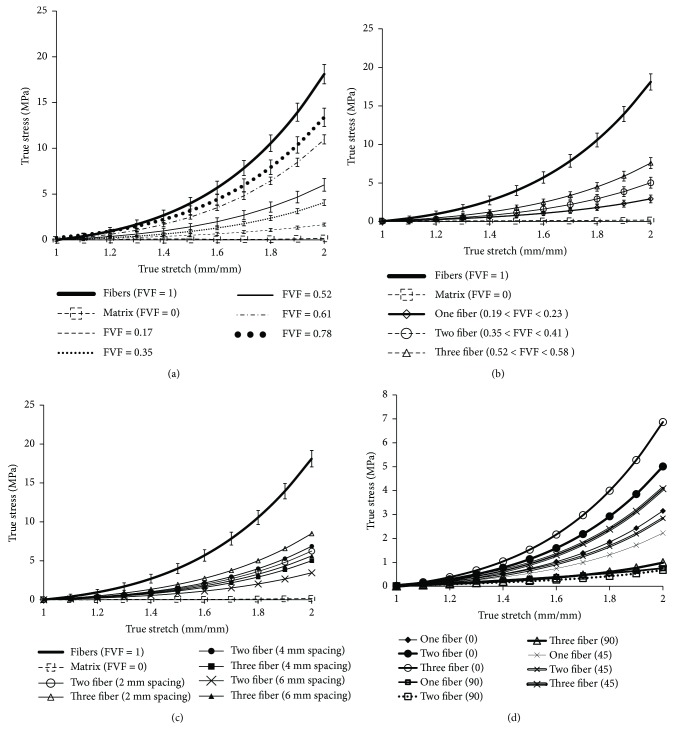
Soft composite material properties due to variations in (a) FVF, (b) number of fibers, (c) fiber spacing, and (d) fiber angle.

The effect of macroscopic FVF on the mechanical properties of the soft composite materials was investigated. A baseline stress-versus-stretch plot of the pure matrix material (composite with no fibers) and a pure fiber material (composite with no matrix) were plotted as shown in Figure 5(a). The stress-versus-stretch plots of the soft composite specimens with FVF of 0.17, 0.35, 0.52, 0.61, and 0.78, respectively, were plotted along with the baseline plots. Each type of specimen was tested three times to ensure repeatability. It was found that the mechanical behavior of all the composite specimens lies within the bound of the baseline plots. Also, with an increase in FVF, the soft composite material exhibited a stiffening behavior. Stiffening behavior has been observed earlier by Annaidh et al. [12] in their macroscopic testing of skin tissue from different parts of the body and has been explained as due to the possible alignment of microscopic collagen fibers along the direction of loading with increased loading. Also, several multiscale studies on tissues have reported the relation between collagen distribution and macroscopic tissue mechanical properties. However, it should be mentioned here that in the current study, the fibers are purely macroscopic and were fabricated to simulate simplified composite mechanical properties and macroscopic anisotropy. No extrapolations or claims could be made with respect to the relationship in mechanical properties or anisotropy of the currently studied macroscopic composite fibers and the microscopic collagen fibers in tissues. Such relationships need to be investigated in future multiscale studies, along with the study of the complex local distribution of collagen fibers and their macroscopic effects.

The stress-versus-stretch results for the soft composite compositions with varying FVFs (in Figure 5(a)) were substituted into the hyperelastic constitutive models, namely, the Mooney-Rivlin, Humphrey, and Veronda-Westmann, and the curve fit constants were estimated with *R*^2^ values over 0.99 (Table 1). These results were further compared with the literature on the characterization of similar elastomers and soft tissue properties. Specifically, modeling using the Mooney-Rivlin model fitting in brain tissues and representative soft elastomers has yielded parameter values of *c*_1_ (±1*E* − 5 to 6*E* − 4) and *c*_2_ (±1*E* − 5 to 1*E* − 3) [22, 26, 34] which were comparably lower than our soft composites with *c*_1_ (±2*E* − 5 to 5*E* − 3) and *c*_2_ (±5*E* − 6 to 5.5*E* − 3). Similarly, Humphrey model parameters (*c*_1_ = ±3.7*E* − 4 to 2*E* − 2, *c*_2_ = ±1*E* − 3 to 5.5*E* − 1) and Veronda-Westmann model parameters (*c*_1_ = ±1*E* − 4 to 3.8*E* − 3, *c*_2_ = ±1.8*E* − 1 to 3.8*E* − 1) were also higher than the literature values (Humphrey: *c*_1_ = ±3*E* − 4 to 1.2*E* − 2, *c*_2_ = ±1*E* − 3 to 5*E* − 1, and Veronda-Westmann: *c*_1_ = ±5*E* − 5 to 3*E* − 3, *c*_2_ = ±1*E* − 1 to 2.5*E* − 1) [22, 26, 34]. In other literature testing with skin and pelvic tissues, and elastomers with representative stiffnesses, higher Veronda-Westmann parameter values of *c*_1_ (±0 to 11.8*E* − 3) and *c*_2_ (±1*E* − 1 to 5*E* − 1) were reported [18, 21, 27, 28, 34, 35], which is expected as the current composite mechanical properties were inferior to skin and close to pelvic tissues (discussed in Section 3.5). It should be mentioned here though that in future studies, using transversely isotropic models in place of isotropic models used in the current work may improve the accuracy of hyperelastic modelling results.

### 3.2. Effect of Fiber Spacing and Number of Fibers on Soft Composite Mechanical Properties

Soft composites with one, two, and three fibers, respectively, were tested, and their mechanical behavior was compared. The average FVF for the specimens with one, two, and three fibers was estimated to be in the ranges of 0.19–0.23, 0.35–0.41, and 0.52–0.58, respectively. The true stress-versus-true stretch plots for all these three configurations are presented in Figure 5(b) along with the baseline plots of specimens with only fibers and only matrix material. As expected, the three-fiber configuration was found to have the stiffest stress-versus-stretch plot compared to the configurations with the one and two fibers. It should be emphasized that these findings may seem intuitive, but to date, they have not been reported for any soft tissue composite material system.

The effect of spacing between the fibers on the soft composite material properties was investigated. For the two- and three-fiber configurations, a spacing of 2 mm, 4 mm, and 6 mm was incorporated, respectively. The results are shown in Figure 5(c); based on which, three main observations could be noted. First, the soft composite material becomes more compliant with increased fiber spacing. Second, the two-fiber configuration with a small fiber spacing (2 mm) is stiffer than the three-fiber configuration with a huge fiber spacing (6 mm). Thus, it can be concluded that fiber spacing can be altered to generate material properties superior (in terms of stiffness) than in the case of more fibers or with a higher FVF as observed earlier in Figures 5(a) and 5(b). Third, the three-fiber configuration with the minimum fiber spacing (2 mm) was found to generate the stiffest soft composite material, and the two-fiber configuration with the maximum fiber spacing (6 mm) was the most compliant material model. It can be concluded from this observation that the maximum number of fibers with the minimum fiber spacing is the combination for obtaining the stiffest soft composite material and the reverse relation is applicable for obtaining a highly compliant soft composite material.

### 3.3. Effect of Fiber Orientation on Soft Composite Mechanical Properties

The effect of fiber orientation on the soft composite mechanical properties was studied for the longitudinal (0 degree), transverse (90 degree), and the ±45-degree fiber orientations. All the angles were measured relative to the direction of application of load (during the mechanical testing). The main observation (from Figure 5(d)) was that the longitudinal fiber orientation with the maximum number of fibers yielded the stiffest composite material. The transverse fiber orientation was found to generate the weakest soft composite materials. For a particular number of fibers (one, two, or three), the ±45-degree fiber orientation led to composite material properties in between the longitudinal and transverse configurations. The combined effect of the number of fibers and the fiber orientation, however, indicated some interesting results. First, the ±45-degree orientation with three fibers was found to be stiffer than the longitudinal configuration with one fiber. It can be thus concluded that the skewed configurations with excess fibers can have composite material properties similar to longitudinal composite configurations with a few fibers. Second, increasing the number of fibers minimally affects the mechanical behavior of composites with transverse configurations (as can be observed in Figure 5(d)). Also, such observations correlate to the fact that tissues with collagen fibers oriented along the principal direction are the stiffest and the ones with fibers along the transverse direction are the weakest [12], with any other orientation causing material behavior to lie in between that in case of the longitudinal and transverse cases.

### 3.4. Combined Effects of FVF and Fiber Orientation on Soft Composite Mechanical Properties

**Figure 6:**
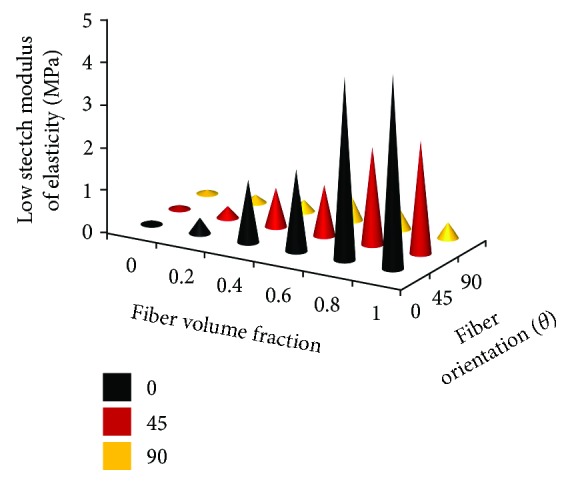
Combined effects of FVF and fiber orientation on low stretch modulus values of soft composite materials.

In soft tissues, the main measurable quantities using imaging, dissection, and histological techniques are the regional FVF and the fiber orientation. Thus, to capture the combined effect of these two parameters, the modulus of elasticity of the various test specimens was estimated at low stretch values. The way this was implemented was by drawing a tangent to the nonlinear stress-versus-stretch plot of any specimen starting at the origin and measuring its slope [12]. The trends observed are summarized in the 3D plot in Figure 6. It can be seen clearly that a high FVF and fiber orientation close to zero degrees generate the stiffest soft composite materials. Also, the transverse fibers have a negligible effect on the composite material properties studied under a uniaxial tension test. This is not a realistic case though, and in a biaxial test may indicate different trends. It should be mentioned here that one of the limitations of our work is that no biaxial or multiaxial tests were conducted to simulate real-life tissue load responses, and such studies will be conducted in future. The 3D plot indicates the options with the FVF and fiber orientation combinations which will generate a certain type of tissue anisotropy model with a specific low stretch modulus of elasticity value.

### 3.5. Comparison of Soft Composite Mechanical Properties with Human Tissues

**Figure 7:**
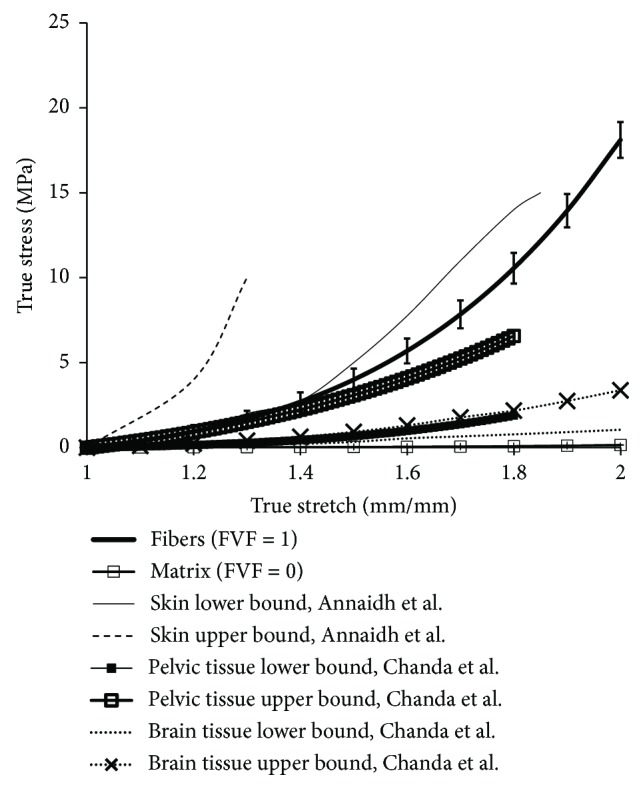
Comparison of the range of soft composite material properties with that of the human skin [12], pelvic [20], and brain tissues [26].

The bounds of the soft composite material properties were compared with that of the material behavior of major human tissues, namely, the skin, pelvic tissues, and brain tissues to emphasize the broad scope of the current work. As seen in Figure 7, the pelvic and brain tissues could be easily recreated using the soft composite material model by adjusting the FVF, fiber orientation, and fiber spacing individually or in combination as shown in the previous sections. A stiffer composite material model needs to be adopted to simulate the anisotropic human skin tissues, as the stress-versus-stretch ranges are much higher. In the future, more sophisticated modeling of soft tissue composites will be attempted to precisely mimic the anisotropic material properties of these three tissue types and also other tissues which are part of the human body.

## 4. Conclusions

In the current work, a soft composite material model was developed experimentally using elastomer-based materials to model tissue anisotropy. A hard elastomer material was used for the fibers and embedded into a soft matrix material to fabricate the soft composite materials. The fiber volume fraction (FVF), fiber orientation, number of fibers, and the fiber spacing were varied to study their effects (stress-versus-stretch response) on the soft composite mechanical behavior. FVF changes were found to most significantly affect the soft composite mechanical properties followed by the fiber orientation and spacing variations. An elasticity modulus term at low stretch ratios was defined, and its variations due to changing FVF and fiber orientations were tracked, similar to soft tissues, where the collagen fiber FVF and fiber orientations are the main drivers of anisotropic behavior. Also, the bounds of the mechanical properties of the developed soft composite materials were compared with some of the major human tissues, namely, the skin, pelvic tissue, and brain tissues to understand how to engineer such soft tissue material surrogates. Additionally, the stress-versus-stretch plots of the composites with varying FVFs were characterized using three well-known nonlinear hyperelastic constitutive material models, namely, the Mooney-Rivlin, Veronda-Westmann, and Humphrey models, and compared with the literature results in soft tissues and elastomers with similar representative properties.

In this study, the soft composite material system is novel and lays the foundation ground for further research in the area of tissue anisotropy modeling. In the future, biaxial and multiaxial testing with such materials will allow researchers to delve into regional tissue properties in human organs and possibly allow a better understanding of their biomechanics. Also, the current work could be used as a baseline for devising various tissue phantoms for biomechanical testing of tissues in normal and diseased conditions. A few limitations of the study should be acknowledged. Even though this macroscopic material system is able to characterize the mechanical tissue anisotropy, it is far from mimicking the actual microscopic structure of a tissue. In the future, additive manufacturing techniques may be able to more realistically capture fiber distribution and orientations in different layers within a tissue. Also, soft composite mechanics was not characterized in this study for different strain rates, which is important to understand the load response of tissues under impacts. Additionally, the use of transversely isotropic hyperelastic models for soft composite characterizations may be more accurate over the isotropic hyperelastic models used in this work, for applications in computational modelling.

## Figures and Tables

**Table 1:** Hyperelastic curve fit coefficients for the soft composite materials developed with varying FVFs (presented in Figure 5(a)).

Fiber volume fraction (FVF)	Mooney-Rivlin		Veronda-Westmann		Humphrey	
	*c*_1_	*c*_2_	*c*_1_	*c*_2_	*c*_1_	*c*_2_
1.00	5*E* − 3	−5.5*E* − 3	3.8*E* − 3	3.7*E* − 1	1.6*E* − 3	5.55*E* − 1
0.00	2*E* − 5	5*E* − 6	1*E* − 4	1.8*E* − 1	2*E* − 2	1*E* − 3
0.17	5.6*E* − 4	−6.1*E* − 4	5.9*E* − 4	2.8*E* − 1	3.7*E* − 4	3.4*E* − 1
0.35	1.2*E* − 3	−1.3*E* − 3	9*E* − 4	3.6*E* − 1	5.4*E* − 4	4.4*E* − 1
0.52	2.4*E* − 4	−9*E* − 5	1.5*E* − 3	3.88*E* − 1	9*E* − 4	4.2*E* − 1
0.61	3.4*E* − 3	−3.9*E* − 3	2.5*E* − 3	3.55*E* − 1	1.2*E* − 3	5*E* − 1
0.78	3.8*E* − 3	−4*E* − 3	3*E* − 3	3.6*E* − 1	1.7*E* − 3	4.6*E* − 1

## Data Availability

The data used to support the findings of this study are available from the corresponding author upon request.
